# Quantitative correlation of mechanoreceptors in tibial remnant of ruptured human anterior cruciate ligament with duration of injury and its significance: an immunohistochemistry-based observational study

**DOI:** 10.1186/s10195-018-0498-7

**Published:** 2018-07-18

**Authors:** Mayur Nayak, H. L. Nag, Sahil Gaba, T. C. Nag, Saurabh Sharma

**Affiliations:** 1 Department of Orthopaedics, All India Institute of Medical Sciences (AIIMS), New Delhi, 110029 India; 2 Department of Anatomy, All India Institute of Medical Sciences (AIIMS), New Delhi, India; 3 All India Institute of Medical Sciences (AIIMS), Jodhpur, India

**Keywords:** Anterior cruciate ligament (ACL), Proprioception, Mechanoreceptors, Immunohistochemistry, Neurofilament protein (NFP)

## Abstract

**Background:**

Proprioception is a specialized sensory modality encompassing the movement of the joint and its position in space. Reconstruction of the anterior cruciate ligament (ACL) does not always yield expected outcome, suggesting that successful reconstruction depends on not only the ultimate strength of the graft but also recovery of proprioception. Treatment delay is a significant concern in developing countries, e.g., in Asia. Thus, presence of mechanoreceptors is one of the factors having paramount importance for successful outcome. We conducted this study to identify mechanoreceptors via immunohistochemical staining and correlate their presence with duration of injury.

**Materials and methods:**

A total of 38 injured native ACL stumps were harvested from patients undergoing ACL reconstruction and stained with neurofilament protein stain to detect functional mechanoreceptors.

**Results:**

Of the specimens, 44.7% stained positive for monoclonal antibody. No association was found between duration of injury and presence of mechanoreceptors (*p* = 0.897). No correlation was seen between age and side.

**Conclusions:**

No correlation was found between duration of injury and presence of viable mechanoreceptors, hence it is beneficial to preserve the native ACL stump irrespective of the time interval between injury and surgery.

**Level of Evidence:**

III.

## Introduction

Proprioception is a specialized sensory modality encompassing the movement of the joint and its position in space. Presence of mechanoreceptors in the anterior cruciate ligament (ACL) has been well documented in the past by various authors [1–6]. Thus, the ACL functions as a sensory organ, providing proprioception and stabilizing muscular reflexes while walking. ACL deficiency leads to instability, thus disabling the daily life of an individual [7–9]. Reconstruction of the ACL does not always yield expected outcome, suggesting that successful reconstruction depends on not only the ultimate strength of the graft but also recovery of proprioception [10–13]. A few authors have tried to quantify the proprioceptive potential of the injured ACL stump. Denti et al. [14] analyzed 20 injured human ACL stumps using gold chloride stain and found that normal mechanoreceptors persisted for 3 months after injury. After that, there was a gradual decrease in the number of mechanoreceptors (one after 9 months and none after 1 year). However, Dhillon et al. [15] and Georgoulis et al. [16] reported persistence of mechanoreceptors in the ACL stump as late as 42 months and 3 years postinjury, respectively.

Various authors have demonstrated growth of mechanoreceptors in the reconstructed ACL. Barrack et al. [17] found an increase in the mechanoreceptors in the ACL graft 6 months after surgery as compared with the normal patellar tendon in canines. Denti et al. [14] found mechanoreceptors in bone patellar tendon autograft in sheep knees 3 months after implantation, and also in two lax human knees with failed semitendinosus autografts 9 and 10 years after surgery, leading to the conclusion that the ACL remnant is a possible source of neural reinnervation of the graft.

Shaving off the ACL remnant helps improve visualization of landmarks over the tibia and femur and makes surgery technically simpler [18]. Also, preservation of the remnant has been shown to be a risk factor for development of arthrofibrosis and cyclops lesion of the knee [15, 16]. Thus, preservation of the ACL stump has both advantages and disadvantages. We evaluated the proprioceptive potential of the ACL stump in human knees with complete ACL tear using mononuclear antibodies to neurofilament protein (NFP) and correlated the results with duration of injury.

## Materials and methods

Patients younger than 50 years of age with symptomatic ACL tear were included in this study. All underwent arthroscopic ACL reconstruction with autologous hamstring graft. History of previous knee surgery, tissue with morphological signs of degeneration or inflammatory disorder, lacerated or crushed stumps which precluded adequate sampling, multiligamentous injury, and failure to obtain consent were the exclusion criteria. The study was approved by the Institutional Review Board and Ethics Committee. Ethical standards according to the Helsinki Declaration of 1964 (and its later amendments) were conformed to. Informed consent was obtained from all patients.

A total of 43 patients with symptomatic ACL tear were enrolled in the study, of whom 38 met our inclusion criteria. Three patients had scarred stumps from which adequate sampling could not be done, one patient was diagnosed as a case of ankylosing spondylitis, and one patient had posterior cruciate ligament (PCL) tear on diagnostic arthroscopy. Intraoperatively, residual ACL tissue was harvested by cutting stumps from as near to the attachment site as possible using arthroscopic scissors. When ACL was adhered to PCL, it was carefully dissected off the PCL then resected.

### Immunohistochemical staining

All received specimens were fixed in 4% formalin phosphate-buffered saline (PBS) and paraffin embedded by routine procedure. Multiple four-micron-thick tissue sections were cut from these paraffin blocks. Slides were prepared and deparaffinized.

#### Primary antibody

Neurofilament-H protein (200 kDa) (lysine–serine–proline repeat) antibody (Chemicon, USA) at dilution of 1:1000 was used as the primary antibody.

#### Secondary antibody

Peroxidase conjugate was used as the secondary antibody.

#### Steps

Multiple sections (>100 per sample on average) were obtained from each specimen and stained immediately to preserve their antigenicity. All were subjected to indirect immunoperoxidase method of staining. Cut sections were mounted on freshly prepared 0.01% poly-l-lysine-coated slides. The samples were dried overnight at 37 °C, dewaxed in xylene, and dehydrated. Hydrogen peroxide (0.3%) in methanol was added for 10 min, and endogenous peroxidase activity was blocked, followed by three washes in PBS. Slides immersed in citrate buffer were put in a microwave for one cycle of 10 min. The slides were then brought to room temperature and immersed in PBS. The primary antibody (which stains NFP) was then applied in optimal dilutions for 1 h and 30 min in a moist chamber, followed by three washes in PBS for 5 min each. Following this, the sections were covered with freshly prepared diaminobenzidine (DAB) solution for 5 min and rinsed with water. Counterstaining was done with hematoxylin for 1 min, followed by one wash in Scott’s tap water, then the sections were dehydrated, cleared with xylene, and mounted with DPX (a mixture of distyrene, a plasticizer, and xylene).

### Interpretation of histology

The prepared slides were reviewed by two independent histologists in blinded fashion. The whole length of the slice was carefully checked. All recordings were manual. To avoid multiple counting of the same receptor, characteristic structure surrounding the mechanoreceptors was noted and identified. Initial screening was done under power of 10×, and areas containing NFP-positive nerve fibers were magnified again to power of 40×. Numerous NFP-positive nerve fibers were typically found in bundles located around the synovium and in interfascicular gaps near the insertion site. Most fibers were found near blood vessels. Examination of serial slices was carried out to correctly identify mechanoreceptors, as they have relatively large size.

### Statistical analysis

Quantitative data are described by mean and standard deviation with 95% confidence interval. Chi-squared test was applied to qualitative data. Univariate logistic regression analysis was carried out to calculate the odds ratio. The receiver operating curve was used to calculate the cutoff point. All statistical tests were two-tailed, with *p*-value <0.05 being taken as significant.

## Results

Of the patients, 36 were male and 2 were female. Mean age was 27 years (range 18–39 years, SD 7.21 years). Mean duration of injury was 22 months (range 0.25–120 months, SD 27.74 months). On arthroscopic evaluation, 22 had medial meniscus tear, 12 had lateral meniscus tear, and 6 had both. In 14 knees, the ACL stump was found adhered to the PCL and was carefully separated before harvesting. In four knees, the ACL stump was adhered to the tibial articular surface posterior to the tibial footprint, and 20 were free. In 17 cases, mechanoreceptors were identified, as summarized in Table 1.

**Table 1:** Frequency, morphology, and location of mechanoreceptors in tibial remnant of ACL

Case number	Injury–surgery interval (months)	ACL stump pattern	Position	Number of MRs
3	2	Free	IF	1
5	12	Adhered to PCL	ST	2
7	78	Adhered to tibia	ST	1
11	6	Adhered to PCL	ST	3
12	6	Free	ST	1
17	60	Adhered to PCL	IF	1
18	12	Free	ST	7
21	36	Adhered to PCL	IF	1
26	24	Adhered to PCL	IF	2
29	48	Adhered to PCL	IF	1
30	12	Adhered to PCL	IF	1
31	32	Adhered to PCL	ST	1
33	9	Free	ST	3
35	4	Free	IF	1
36	9	Adhered to PCL	ST	3
37	18	Adhered to PCL	ST	9
38	16	Adhered to tibia	IF	1

*MR* mechanoreceptor, *IF* interfascicular, *ST* subsynovial tissue

### Quantitative evaluation of mechanoreceptors

**Fig. 1:**
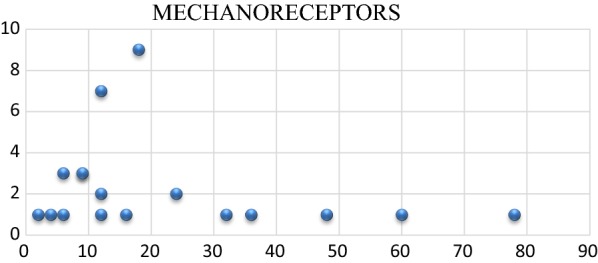
Scatter plot showing distribution of number of mechanoreceptors (*X*-axis) versus duration in months (*Y*-axis). There was no significant correlation between total number of mechanoreceptors and interval from injury to operation

**Fig. 2:**
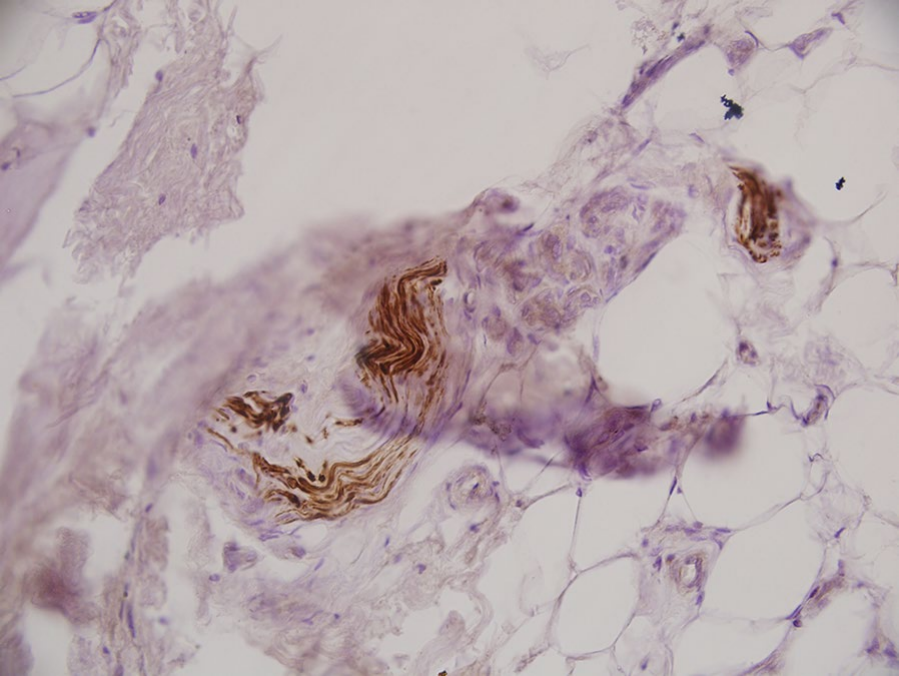
Light microscopy (power 40×) showing NFP-positive slender axons

Monoclonal antibody staining of nerve fibers was positive in 44.7% (17 out of 38) of the specimens. To assess the correlation between duration of injury and presence of mechanoreceptors, univariate logistic regression was carried out and the odds ratio calculated. The odds ratio was 1.008. There was no association between duration of injury and presence of mechanoreceptors (*p* = 0.897) (Fig. 1). The receiver operator curve (ROC) was plotted to determine the cutoff period for the stump before which viable proprioceptor would have been found. The area under the curve recorded was 58.68%, which was nonsignificant. No correlation was seen between age and side of injury. As there were only two females (much smaller than the number of males), the relationship between sex and the number of mechanoreceptors cannot be commented upon. Meniscal tear did not have any significant relationship with any of the outcomes. Results were analyzed using Strata software version 14.0. Figure 2 (light microscopic image at high power of 40×) shows neurofilament protein (NFP)-positive slender axons (mechanoreceptors).

## Discussion

Proprioception of the knee is an important consideration for successful ACL reconstruction. The maximum number of nerve endings reside at the attachment site of the ligament to the bone, which serves as the main tract for proprioceptive feedback [14]. Few studies have focused on the presence of mechanoreceptors in the torn ACL stump [2, 12, 14–17, 19] or its role as a source of neural innervation of the graft. However, some studies have shown that leaving the ACL stump has its own disadvantages such as formation of cyclops lesion, arthrofibrosis, and difficulty in identifying anatomical landmarks during surgery [15, 16].

Three studies employing gold chloride staining to look for mechanoreceptors have been conducted to date [14, 16, 19]. Denti et al. [14] found that mechanoreceptors were detectable up till 3 months after injury, after which they gradually decreased in number. They did not find any mechanoreceptors in any case with duration of injury of more than 1 year. Adachi et al. [19] performed a similar study and found mechanoreceptors in all examined specimens (with duration of injury ranging from 2 months to 10 years). They concluded that there is no correlation between the number of mechanoreceptors and duration of injury, in contradistinction to the finding of Denti et al. Georgoulis et al. [16] found mechanoreceptors in all cases where a portion of the ACL was attached to the PCL, but none or very few in stumps which were free. The shortcomings of gold chloride staining must be acknowledged. Firstly, there is a lack of standardization of the staining technique [2, 3, 23]. Secondly, nonspecific staining of blood vessels, collagen, and elastic fibrils may occur, resulting in incorrect labeling as mechanoreceptors [20, 23]. Third, mechanoreceptors picked up by this method may be morphologically normal while their significance as far as proprioception is concerned is equivocal [1, 15].

**Fig. 3:**
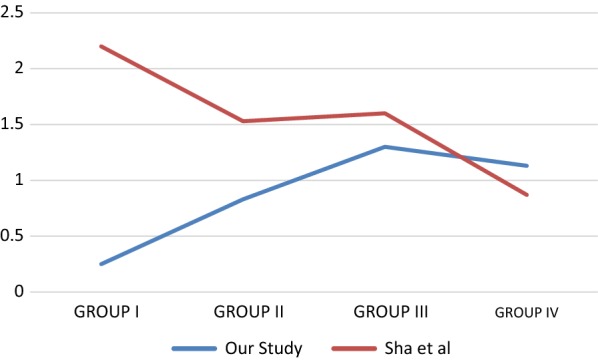
Comparison of number of mechanoreceptors over a graded time period for our study versus that of Sha et al. [22]

In the study conducted by Sha et al. [21], 60 patients undergoing arthroscopic ACL reconstruction were divided into four groups depending upon duration of injury (group I, no more than 3 months; group II, 3–6 months; group III, 6 months to 1 year; group IV, more than 1 year). No significant difference was found among groups I–IV, however the overall trend was for a decrease in the number of mechanoreceptors with the passage of time. Creating similar timeframes for our patients, Fig. 3 shows graphically the difference between the results of the two studies. Although our study shows a trend opposite to that of Sha et al., the increase is statistically insignificant.

Lee et al. [2] used the same immunohistochemical method as applied here, finding 17 mechanoreceptors in 12 out of 36 specimens compared with 19 mechanoreceptors in a control group of 2 specimens. Five of them had necrotized morphology with weak staining and loose form, which was often found in degenerative joint. They speculated that the receptors probably degenerated after injury. The number of receptors was not presented versus duration of injury by Lee et al. In our study, we did not find any mechanoreceptors with necrotic morphology, and 17 out of 38 specimens were positive for mechanoreceptors on the scale of varying time frame.

Dhillon et al. [15] conducted a similar study, where the reactivity to NFP monoclonal antibody for mechanoreceptors in ruptured ACLs was 52.4%, quite similar to the value of 44.7% found in the present study. In contrast to our results, they found a statistically significant relationship between shorter duration of injury and higher proprioceptive potential of injured ACL stump.

Gao et al. [22] found a significant decrease in the number of mechanoreceptors in the ACL stump with increasing duration of injury, which is different from our study. However, they used S-100 instead of NFP staining.

The inconsistency between our and previous studies might be due to variations in specimen collection, sectioning, and processing. First of all, complete harvesting of the specimen was done as close to the attachment as possible in our study, as opposed to some other studies in which the whole residual stump was not harvested. Secondly, most of the other authors using indirect methods applied the S-100 staining method [1, 15, 21, 22]. This method has been proved to stain free nerve endings significantly, but whether these nerve endings have any proprioceptive function remains to be determined [1]. On the other hand, we used monoclonal antibody to NFP, which stains proprioceptive axon cylinders. The validity of this method has been proved by Bali et al. [1]. This also explains the lesser percentage of positive cases in our study compared with previous studies.

The mechanoreceptor detection rate was highest in cases where the ACL stump was attached to the PCL (10 out of 14 cases; 71.4%), whereas the positivity rate was lower in free stumps (5 out of 20 cases; 20%) or where the ACL stump was attached to the tibia (2 out of 4 cases; 50%). This may reflect the higher volume of stump harvested from cases with PCL-adherent stump [15].

Treatment delay is a significant concern in developing countries such as ours. One of the issues that still sparks debate is the persistence of viable mechanoreceptors in the ACL stump and its correlation with duration of injury. Studies have proved that there is a decrease in the mechanoreceptors with increase in duration of injury [14, 18], which may adversely affect the surgical outcome. Mechanoreceptors in the ruptured ACL have also been proposed to play a role in restoring joint stability. Additionally, preserving the native ACL stump can accelerate the cellular proliferation and revascularization of the grafted tendon and improve its biomechanical strength.

### Strengths of the study

This was an observational study of human samples having a wide timeframe following injury. Moreover, an indirect method for detection of nerve fibers using immunohistochemistry was applied rather than direct methods, which may increase the yield.

### Weaknesses of the study

The weaknesses of this study include small sample size, no correlation with clinical outcomes, and the lack of data regarding extent of reinnervation on follow-up after ACL reconstruction.

The results of this study indicate that there is no correlation between duration of injury and presence of viable mechanoreceptors. Thus, it is beneficial to preserve the native ACL stump irrespective of the time interval between injury and surgery. This observation has a potential bearing on the surgical outcome, as preservation of the native ACL stump may be a source of reinnervation of the ACL graft. Also, the probability of finding mechanoreceptors is greater if the ACL stump is attached to the PCL. Further studies are required on the regeneration of mechanoreceptors in the grafted tendon to validate these findings.
